# Effect of Lipase and Lysolecithin Supplementation with Low Energy Diet on Growth Performance, Biochemical Attributes and Fatty Acid Profile of Breast Muscle of Broiler Chickens

**DOI:** 10.3390/ani13040737

**Published:** 2023-02-18

**Authors:** Aziz Ahmad, Gulfam Ali Mughal, Rani Abro, Shamsuddin Bughio, Huma Rizwana, Imdad Hussain Leghari, Shoaib Ahmed Pirzado

**Affiliations:** 1 Department of Animal Nutrition, Sindh Agriculture University, Tandojam 70060, Sindh, Pakistan; 2 Department of Veterinary Pharmacology, Sindh Agriculture University, Tandojam 70060, Sindh, Pakistan; 3 Department of Livestock Management, Sindh Agriculture University, Tandojam 70060, Sindh, Pakistan; 4 Department of Poultry Husbandry, Sindh Agriculture University, Tandojam 70060, Sindh, Pakistan

**Keywords:** lysolecithin, lipase, production performance, nutrient retention, fatty acid profile, broiler

## Abstract

**Simple Summary:**

The exogenous enzymes and emulsifiers have the potential to produce more energy from fat and improve its utilization and absorption by poultry birds. Therefore, the main goal of this study is to investigate the effects of ditary supplementation of lipase and lysolecithin on growth performance, nutrient digestibility, and meat quality parameters of broilers chicken fed low energy diet. From our findings, the supplementing 0.08% lipase and lysolecithin with a low-energy diet could exhibit better growth performance and meat quality of broilers. Moreover, lipase and lysolecithin alleviate the negative effect of low energy in broilers

**Abstract:**

This study aimed to investigate the effect of dietary lysolecithin (LYSO) and lipase supplementation on productive performance, nutrient retention, and meat quality of broiler chicken fed a low energy diet. For this purpose, a total of 360 chicks were randomly alienated into six treatments, having six replicates (no = 10) birds each replicate. The dietary treatments were followed as control (CON fed as normal energy diet), LE (CON—100 kcal/kg from BD. basal diet), LIP 0.04 (LE + 0.04% lipase), LYSO 0.04 (LE + 0.04% lysolecithin), LIP + LYSO 0.04 (LE + 0.04% lipase and lysolecithin), and LIP + LYSO 0.08 (LE. + 0.08% lipase and lysolecithin). The birds fed with LIP + LYSO 0.04 exhibited higher weight gain than LYSO 0.08 and CON (*p* < 0.05), and higher feed intake (F.I.) was also observed in LIP + LYSO 0.04 than CON. However, lipase and emulsifier dietary effects were non-significant on FCR. (*p* > 0.05). Effects of experimental diets on dry matter (DM), crude protein (CP), and fat digestibility were also non-significant (*p* > 0.05). Similarly, the blood biochemical profile (total cholesterol, triglycerides, LDL, HDL) of the broiler showed no significant difference (*p* > 0.05) by dietary treatments. Similarly, liver enzymes, AST and A.L.T., were also not statistically significant (*p* > 0.05) among all dietary treatments. Similarly, supplementation of LIP and LYSO had a non-significant (*p* > 0.05) effect on breast meat fatty acids composition. Conclusively, adding LIP + LYSO 0.08 to a low energy diet could demonstrate better growth performance and reduce the negative impact of a low-energy diet.

## 1. Introduction

In modern poultry feed formulation practices, fat addition is one of the most common strategies to fulfill broilers’ high energy demand, as fat provides almost double the energy compared to carbohydrates and proteins. Two types of oil sources, i.e., vegetable oils and animal fats, are used in commercial broiler diets to improve dietary energy and hence the growth performance of broilers [1,2]. However, increasing dietary oil levels can adversely affect digestibility [3,4], particularly in young broiler chickens [5]. The poor digestibility of fats in young broilers is due to the poor duodenal secretion of lipase and reduced synthesis of bile salts [6,7,8]. The physiological limitations of utilizing fats in broilers can be overcome by adopting endogenous and exogenous strategies. First, emulsifier supplementation (bile salts, lysolecithin, or lysophospholipid-ids) can improve lipid digestibility by increasing the active surface of lipid droplets and stimulating micelle formation [9,10,11]. Second, exogenous lipase might be incorporated into poultry feed to enhance fat digestion [12]. Low-density diets of broilers containing exogenous enzymes have been found to exert positive effects on growth performance [13,14].

The dietary addition of lysophospholipids improves weight gain, nutrient digestibility, and better economics in broilers [15]. Moreover, lysolecithin, an addition to the broiler diet, also improved nutrient and energy utilization in young broilers [16]. Additionally, soy-lecithin alone or in combination with lipase improves broilers’ weight gain and antioxidant capacity [17]. Other researchers [9,18,19] also found positive effects of feeding diets containing emulsifiers in broilers with low-energy diets. Previous studies have also reported feeding diets containing emulsifiers with improved performance in broilers. Contrary to this, ref. [20] has noticed no significant effect on the growth performance of broilers after supplementation of low energy density diet with emulsifier and lipase. Similar observations were also indicated by [21,22,23,24] after the addition of lipase and emulsifiers either in combination or alone in broilers. After feeding low energy diets with emulsifiers, improved dry matter and crude protein digestibility were observed in broilers [24]. However, ref. [25] stated that supplementation of lipase increases the growth performance of broilers fed low energy diets. However, ref. [26] observed no effect of high energy diet on blood serum parameters. Besides, ref. [26,27,28] reported that high energy diets and low-energy diets had non-significant effects on blood serum parameters and meat quality parameters of breast and thigh meat, such as pH and water-holding capacity (WHC), respectively.

As there is a paucity of data regarding emulsifier and/or lipase supplementation in broiler diets, the purpose of this study was to explore the effect of supplementing lipase and emulsifier with low dietary energy on broiler growth performance, blood profile, and breast meat fatty acid profile.

## 2. Materials and Methods

### 2.1. Experimental Design and Feeding Management

The proposed experimental trial was conducted at the Poultry Experimental Station, Department of Animal Nutrition, Sindh Agriculture University, (SAU), Tandojam, Pakistan. All the methods and protocols were approved by the animal care and ethics committee of SAU, Tandojam, Pakistan. A total of 360 one-day-old chicks were randomly assigned to six treatment groups, with each replicate containing ten birds. Two-phase diets were formulated, i.e., starter (0–21 days) and finisher (22–35 days). The dietary treatments were as follows: control (CON fed as normal energy diet), L. (CON—100 kcal/kg from B.D., basal diet), LIP 0.04 (LE + 0.04% lipase), LYSO 0.04 (LE + 0.04% lysolecithin), LIP + LYSO 0.04 (LE + 0.04% lipase and lysolecithin), and LIP + LYSO 0.08 (LE + 0.08% lipase and lysolecithin). The experimental shed was cleaned and disinfected before the arrival of the chicks. The trial was conducted on a wood shaving-covered floor; temperature was maintained during the first week at 34 °C and gradually declined at 2 °C every week until it reached 24 °C. Initially, 23 h lighting and one-hour darkness was provided, followed by 23, 21, 19, and 18 h lighting for the 2nd, 3rd, 4th, and 5th week, respectively. The birds were provided ad-libitum access to feed and water. The ingredient composition, calculated level, and analyzed value of experimental diets are presented in Table 1.

### 2.2. Growth Performance

The growth performance of birds was recorded by measuring their body weight at days 21 and 35 on a replicate basis. Feed intake (F.I.) and feed conversion ratio (F.C.R.) were calculated.

### 2.3. Nutrient Digestibility

On their 32nd day of age, three birds per replicate were transferred to a separate pen for excreta collection. Birds were given a one-day adaptation period. Fecal excreta samples were collected for three days in a row, from the 33rd to the 35th. The feces excreta were dried in an oven at 65 °C for 72 h over two days. The dried fecal samples were ground, and the nutritional digestibility was determined. The apparent nutrient digestibility coefficients were calculated using a method developed by [29].

Digestibility% = (Nutrient intake − Nutrient excreted)/(Nutrient intake) × 100

### 2.4. Meat Quality Parameters

After slaughtering, breast and thigh meat samples were collected and stored at 4 °C. For the estimation of pH, 1.5 g of grinded meat was taken and homogenized in 10 mL of water. The triplicate readings of each sample were assessed using a pH meter (PCE-228, PCE instruments pvt L.T.D., Nordrhein-Westfalen, Germany). The pH was measured on 2 and 24 h of post-slaughtering. Then, 15 g of meat from the thigh and breast were weighed individually, placed in zipper plastic bags, and put in warm water at 75 °C. Drip loss was determined by using the described method [30]. Water-holding capacity (W.H.C.) was examined by using a previously-described procedure [20]. To determine WHC, 10 g of grinded sample were taken, added with 12 mL NaCl, and centrifuged at 10,000 rpm for 15 min. The supernatant was collected for measurement in a measuring cylinder. The change in the amount of NaCl solution that had been consumed was 0.6 M, and the supernatant was WHC.

WHC (%) = (Original weight − Supernatant)/(Original weight) × 100(1)

### 2.5. Blood Biochemical Attributes

After trial (35th day), blood samples were taken after slaughtering the birds in sterilized B.D.-vacutainers. The serum was obtained by centrifugation at 2500 rpm for 15 min, and the obtained serum was collected in Eppendorf tubes and stored at −20 °C until analysis. The serum level of total cholesterol, Triglyceride, H.D.L. and L.D.L., were analyzed by using automated biochemical analyzer (BTS-350, Biosystem pvt, LTD, Champagne au Mont d’Or, France). The serum for determining ALT and AST enzymes (which are markers of oxidative damage sustained in hepatic tissue) was analyzed by AST Activity Assay Kit MAK 055 and ALT Activity Assay Kit MAK 052 (Sigma-Aldrich, pvt LTD, Schnelldorf, Germany) according to manufacturer’s protocol.

### 2.6. Breast Muscle Fatty Acid Profile

Ten grams of breast meat samples (Pectoralis Major) were taken in tubes and stored in liquid nitrogen (−96 °C) immediately for further analysis. The fatty acid profile of breast meat samples was assessed by applying the procedure described by [31].

### 2.7. Statistical Analysis

The data collected were analyzed through the analysis of variance (ANOVA) technique with the help of SAS 9.1. The significant means were compared through Duncan’s Multiple Range (DMR) [32] and LSD.

## 3. Results

### 3.1. Growth Performance

Supplementation of lipase and lysolecithin in low-energy diet resulted in non-linear effects on the performance of broilers (Table 2). During 0–21 days of the feeding trial, weight gain was numerically higher (*p* = 0.07) in LIP + LYSO 0.08, while feed intake and FCR were not significantly (*p* > 0.05) affected by the experimental diet. During the second phase of the experiment (22–35 days), a non-significant (*p* > 0.05) effect of experimental diets on weight gain, feed intake, and FCR was observed. In the overall experimental period, the birds fed a diet containing LIP + LYSO 0.08 showed a significant (*p* < 0.05) effect on weight gain and feed intake as compared to other experimental treatments. However, FCR was not affected significantly (*p* > 0.05) between all treatments.

### 3.2. Nutrient Digestibility

The effect of different dietary treatments on nutrient digestibility is summarized in Table 3. The digestibility percentage of DM, CP, and fat tended to increase in dietary treatments, while no significant (*p* > 0.05) effect was observed among all treatments.

### 3.3. Biochemical Attributes

The impact of dietary supplementations on TG, HDL, and LDL was non-significant (*p* > 0.05), as revealed in Table 4. Similarly, liver enzymes, ALT and AST, were also not statistically significant (*p* > 0.05) among different dietary treatments.

### 3.4. Meat Quality

Effects of different dietary treatments on breast and thigh meat quality attributes are summarized in Table 5. The supplementation of different dietary treatments showed non-significant (*p* > 0.05) differences in breast and thigh meat drip loss, cooking loss, water-holding capacity, and pH.

### 3.5. Fatty Acid Profile of Breast Meat

The effect of different dietary treatments on breast meat fatty acid profile is given in Table 6. Supplementation of different dietary treatments had a non-significant (*p* > 0.05) impact on breast meat’s fatty acids profile.

## 4. Discussion

### 4.1. Growth Performance

Numerous research studies have examined emulsifiers and lipase with low energy diets using essential nutritional strategies to reduce the feed cost without any adverse effect on growth performance by the feed industry. However, in the current research study, we aimed to clarify the possible impact of adding lipase and emulsifier to the broiler diet with reduced energy on growth performance, meat quality, and fatty acid profile.

The present study reported higher FI and growth performance in treatment groups and lower energy than in CON diets. The reduced feed intake in birds fed a high energy diet (CON) in our study could be elaborated on, as higher dietary energy satisfies the energy demand for the growth of birds. The findings of our study are in accordance with the results of [33], who stated that increasing dietary energy could reduce broilers’ feed intake. However, some researchers reported that weight gain in broilers was decreased by being fed a low energy diet compared to the birds who were fed high energy diet [11,26,27,34]. In the present study, the non-linear effects of supplementing lysolecithin and lipase either alone or combined in a low energy diet. However, improved weight gain and feed intake was observed in birds fed a diet with LIP + LYSO 0.08. Similar findings have been confirmed by [15], who noticed that supplementation of lysophospholipids can improve broiler’s performance, especially when lysophospholipids were supplemented in feed “on top” [23,35]. Moreover, several scientists have also reported positive results by adding lysophospholipid to broiler feeds with reduced fat and oil content [11,36]. However, [37] reported that emulsifier supplementation did not affect feed intake in broilers. Our findings are similar to those reported by [38], who found that the dietary addition of emulsifiers significantly influenced the body weight gain and growth rate of chickens overall FCR in birds. Contrary to our findings, some researchers reported that supplementation of lipase and emulsifier, either combined or alone, had non-significant effects on growth performance in broiler in regards to a lower energy level [21,22,23,24]. Likewise, [39] observed that lysolecithin addition 250 g/t of feed could sustain the performance of broilers fed low-energy diets. [25] found that adding lipase and an emulsifier enhances the growth performance of broilers fed low-energy diets.

### 4.2. Nutrient Digestibility

The effect of different dietary treatments showed improvement, but had statistically non-significant (*p* > 0.05) effects on dry matter, crude protein, and crude fat digestibility in broilers fed 0.08% lipase and an emulsifier in low energy diet. According to some researchers, emulsifier and lipase supplementation improve fat digestion, which leads to increased growth performance and intestinal villus length due to improved dietary digestion and energy efficiency [9,18]. Similarly, Ref. [38] observed an analysis of fatty acid digestibility on day 35 of the study, showing a positive effect of adding emulsifier, with the highest increase noted for polyunsaturated fatty acids. Likewise, digestibility of dry matter, ether extract, and apparent metabolizable energy was increased when broilers were fed diets containing emulsifier [15,16,40]. However, Ref. [18] found improved apparent metabolizable energy digestion; the digestion of dry matter and crude protein was not disrupted when chickens were fed on diets containing emulsifier. The results of our study are supported by previous reports [41], who reported an improvement of nutrient digestibility in broiler diets containing fat emulsifiers and low dietary fat content. Correspondingly, Ref. [42] found that adding a low-energy diet with lysolecithin or sodium stearoyl-2-lactate increased nitrogen retention efficiency. Ref. [24] stated that broiler fed negative control diet (low energy) with emulsifier showed increased retention of DM, CP, and ME compared to other treatments on day 21. Our study is in agreement with [1,4], who reported an increase in nutrient digestibility in broiler chickens with exogenous emulsifiers. However, Ref. [43] found no influence of lipase on the retention of EE or apparent metabolizable energy when added in the diet of young chickens. Similarly, Ref. [27] found that fat digestion was not affected by lipase addition in the low energy diet compared to a high energy diet. In our study, nutrient digestibility was not affected by dietary energy level. Similar results were reported by [39,44]—that low- or high-energy diets had no impact on nutrient retention.

### 4.3. Blood Biochemical Attribute

The impact of various dietary treatments had non-significant (*p* > 0.05) effects on TG, HDL, and LDL in broilers. Similarly, liver enzymes, AST and ALT, were also not statistically significant among different dietary treatments. Similar findings were observed by [24,45], who found that cholesterol, glucose, and triglyceride concentrations did not change in broilers fed a diet containing emulsifier or emulsifier and/or lipase. Our study’s findings are similar to those of [46], who noted no differences in biochemical indicators in broiler chickens fed exogenous emulsifier. Similarly, some investigators presented that adding emulsifier in birds’ feed did not affect TG, TC, and HDL, LDL [11,26,47]. However, in layers, serum triglycerides content due to diet comprising 0.05% emulsifier were decreased compared to those from a high-energy diet without emulsifier [21]. On the other hand, Ref. [46] showed no significant impact in serum TC, LDL, or TG levels after adding the emulsifier. However, Ref. [48] reported serum TC levels and LDL were lowered with lysolecithin, while high levels of HDL and triglycerides (TG5) were increased. Similarly, in a study by [49], plasma TC, HDL, TP, and GLOB were reduced in low-energy diet group without emulsifiers but improved with emulsifier addition. In our study, energy levels did not affect blood biochemical profile. Similar to our findings, Ref. [26] described that high dietary energy does not affect blood serum indicators. Likewise, [11] observed that the levels of TG, TC, HDL, and LDL were not affected by energy levels in broilers.

### 4.4. Meat Quality

In our study, supplementing lipase and emulsifier alone or combined in a low energy diet had a non-significant effect on meat quality parameters in broilers. Similar findings were conveyed by [4]—that the sodium stearoyl-2 lactate-supplemented diet did not affect breast meat pH and drip loss at 24 and 48 h after slaughter. Muscle water is an imperative property of meat quality which eventually contributes to the loss of muscle mass and loss of muscle during cooking. On the contrary, Ref. [4] observed that sodium stearoyl-2 lactate can reduce cooking loss in broilers. In our study, the effect of energy level did not affect meat quality in broilers. Consistent with our results, Ref. [27,28] described that low energy intake had a non-significant effect on breast and thigh pH, WHC values compared to a high energy diet. Supplementing various dietary treatments had a non-significant effect on breast meat fatty acids composition. Similar results have been reported by [50], who noted that supplementation of emulsifier shows no impact on the lipid profile in broilers. However, Ref. [49] observed that palmitic acid, oleic acid, and linoleic acid content of the muscles decreases as energy decreases and is supplemented by emulsifiers.

## 5. Conclusions

Based on our results, it could be concluded that dietary supplementation of emulsifier and lipase at the dose of 0.08% in low energy diets exhibited a positive impact on growth performance and nutrient utilization of broiler chicken fed a low energy diet. Moreover, lipase and emulsifier supplementation mitigated the detrimental effects of a low energy diet on the production efficiency of broiler chicken. The world is facing an economic crisis due to increasing cost of energy-rich ingredients. Our study confirms the concept of using lipase enzyme and emulsifier in low energy diets as an alternative tool to reduce the input cost without any negative impact on production performance of broiler chickens. However, further in-depth research is needed for better understanding of the mechanistic role behind the fat digestion and utilization by the dietary addition of emulsifier and lipase in poultry birds.

## Figures and Tables

**Table 1:** Ingredient and nutrient composition of experimental diets for broiler chicks (as-fed basis).

Items	Phase 1 (d 1 to d 21)		Phase 2 (d 22 to d 35)	
	CON ^1^	LE ^2^	CON ^1^	LE ^2^
Ingredients, %				
Maize	45.50	45.50	60.00	61.00
Rice Polish	5.53	7.61	1.31	2.89
Soybean meal, 45% CP	39.70	39.40	29.93	29.14
Soy oil	4.80	3.00	4.80	3.00
Limestone	1.11	1.18	0.75	0.74
Di-calcium phosphate	1.90	1.87	1.73	1.73
Sodium chloride	0.37	0.38	0.39	0.39
Sodium bicarbonate	0.06	0.06	0.08	0.08
L-Lysine Sulphate	0.42	0.38	0.48	0.49
DL-Methionine, 98%	0.38	0.39	0.32	0.33
L-Threonine	0.13	0.13	0.11	0.11
Vitamin and mineral premix ^3^	0.10	0.10	0.10	0.10
Calculated nutrient composition (%)				
ME (Kcal/Kg)	2900	2800	3100	3000
Crude protein%	22	22	19	19
Ether extract%	7.53	6.04	7.38	5.85
Calcium%	0.99	0.99	0.85	0.85
Available Phosphorous%	0.45	0.45	0.42	0.42
Analyzed nutrient composition (%)				
Dry matter	90.52	89.76	89.91	90.63
Crude Protein	22.35	22.15	19.55	19.31
Ether Extract	7.89	6.24	7.54	6.32
Ash	4.23	4.55	4.31	4.66

^1^ Basal diet, ^2^ Low energy, ^3^ Nutrient level of premix (per kg diet): vitamin A, 15,000 IU; vitamin D3, 5000 IU; vitamin E, 40 mg; vitamin K, 3 mg; vitamin B1, 3 mg; vitamin B2, 12 mg; vitamin B3, 60 mg; vitamin B5, 15 mg; vitamin B6, 4 mg; Biotin, 0.2 mg; Folic acid, 3 mg; vitamin B12, 0.03 mg, choline 1850 mg, Iron, 25 mg; copper, 15 mg; zinc, 100 mg; manganese, 110 mg; iodine, 1 mg; selenium, 0.4 mg, CON = control; LE = low energy.

**Table 2:** Effect of low energy diet and supplementation of emulsifier and lipase on growth performance of broiler chickens.

Items	CON	LE	LIP	LYSO	LIP + LYSO	LIP + LYSO	SEM	*p*-Value
			0.04%	0.04%	0.04%	0.08%		
0–21 days								
BW gain (g)	903 ^b^	955 ^ab^	995 ^a^	986 ^a^	946 ^ab^	1014 ^a^	12.82	0.07
Feed intake (g)	1078	1195	1199	1221	1184	1235	18.27	0.16
FCR	1.19	1.25	1.21	1.24	1.25	1.22	0.01	0.96
22–35 days								
BW gain (g)	1273	1302	1271	1299	1278	1336	9.06	0.2
Feed intake (g)	2058	2234	2229	2190	2189	2260	21.08	0.08
FCR	1.62	1.72	1.75	1.69	1.73	1.69	0.08	0.34
0–35 days								
BW gain (g)	2175 ^b^	2256 ^ab^	2266 ^ab^	2285 ^ab^	2223 ^b^	2350 ^a^	16.86	0.02
Feed intake (g)	3135 ^b^	3429 ^a^	3428 ^a^	3411 ^a^	3388 ^a^	3495 ^a^	29.83	0.003
FCR	1.44	1.53	1.51	1.49	1.52	1.48	0.01	0.68

CON = Basal diet, LE = low energy, LIP = low energy + 0.04% lipase, LYSO = low energy + 0.04% lysolecithin, LIP + LYSO 0.04 = low energy + 0.02% lipase and 0.02% lysolecithin, LIP + LYSO 0.08 = low energy + 0.04% lipase + 0.04% lysolecithin. ^a,b^ Means within a row with different superscripts are significantly different (*p* < 0.05). SEM: Standard error of mean.

**Table 3:** Effect of low energy diet and supplementation of emulsifier and lipase on nutrient digestibility in broiler chickens.

Items	CON	LE	LIP	LYSO	LIP + LYSO	LIP + LYSO	SEM	*p*-Value
			0.04%	0.04%	0.04%	0.08%		
Dry matter %	78.59	78.93	80	81.04	80.94	82.29	0.63	0.56
Crude protein %	81.08	81.8	82.19	82.41	81.84	83.46	0.68	0.97
Crude fat %	76.78	78.48	78.22	79.27	79.29	79.68	0.63	0.85

CON = Basal diet, LE = low energy, LIP= low energy + 0.04% lipase, LYSO= low energy + 0.04% lysolecithin, LIP + LYSO 0.04 = low energy + 0.02% lipase and 0.02% lysolecithin, LIP + LYSO 0.08 = low energy + 0.04% lipase + 0.04% lysolecithin. SEM: Standard error of mean.

**Table 4:** Effect of low energy diet and supplementation of emulsifier and lipase on blood biochemical profile of broiler chickens.

Items	CON	LE	LIP	LYSO	LIP + LYSO	LIP + LYSO	SEM	*p*-Value
			0.04%	0.04%	0.04%	0.08%		
Blood profile								
Cholesterol (mg/dl)	172.33	126.33	150.33	158.33	159.33	139.33	8.14	0.71
TG (mg/dl)	149	149.33	141.33	150	147.67	136.33	5.04	0.97
HDL (mg/dl)	40.67	30.67	36.33	37.67	38.67	32.33	1.57	0.46
LDL (mg/dl)	99	94.33	90.67	86	88.33	73	5.24	0.84
Liver enzymes								
ALT (U/L)	20	21	20.67	22.67	26.67	25	1.24	0.64
AST (U/L)	81.33	91.33	65.67	82	81	93.33	6.28	0.88

CON = Basal diet, LE = low energy, LIP = low energy + 0.04% lipase, LYSO = low energy + 0.04% lysolecithin, LIP + LYSO 0.04 = low energy + 0.02% lipase and 0.02% lysolecithin, LIP + LYSO 0.08 = low energy + 0.04% lipase + 0.04% lysolecithin. SEM: Standard error of mean.

**Table 5:** Effect of low energy diet and supplementation of emulsifier and lipase on meat quality in broilers.

Items	CON	LE	LIP	LYSO	LIP + LYSO	LIP + LYSO	SEM	*p*-Value
			0.04%	0.04%	0.04%	0.08%		
pH after 2 h								
Breast	6.57	6.53	6.65	6.38	6.44	6.43	0.05	0.83
Thigh	5.45	6.57	6.4	6.48	6.42	6.49	0.19	0.69
pH after 24 h								
Breast	6.72	6.53	6.58	6.53	6.57	6.65	0.05	0.83
Cooking loss%								
Breast	29.11	26.44	30.22	26	30	25.55	0.86	0.56
Thigh	34.67	34	32.67	33.56	34.67	32	1.3	0.99
Drip loss% 24 h								
Breast	4.02	4.16	3.47	4.15	4.05	3.37	0.17	0.61
Thigh	4.44	4.5	3.55	4.49	4.11	3.95	0.25	0.86
Drip loss% 48 h								
Breast	1.71	1.76	2.03	1.87	1.73	1.6	0.15	0.96
Thigh	1.85	1.82	2.43	1.81	2.03	1.76	0.16	0.8
WHC%								
Breast	53.8	54.12	53.68	54.75	53.48	55.56	0.44	0.86
Thigh	54.86	54.03	54.49	53.31	56.69	55.01	0.65	0.77

CON = Basal diet, LE = low energy, LIP = low energy + 0.04% lipase, LYSO = low energy + 0.04% lysolecithin, LIP + LYSO 0.04 = low energy + 0.02% lipase and 0.02% lysolecithin, LIP + LYSO 0.08 = low energy + 0.04% lipase + 0.04% lysolecithin. SEM: Standard error of mean.

**Table 6:** Effect of low energy diets and supplementation of emulsifier and lipase on the fatty acid profile of breast muscles in broilers.

Items		CON	LE	LIP	LYSO	LIP + LYSO	LIP + LYSO	SEM	*p*-Value
				0.04%	0.04%	0.04%	0.08%		
Myristic acid	C14:0	1.56	1.7	1.62	1.56	1.65	1.59	0.09	0.98
Palmitic acid	C16:0	20.05	16.74	19.42	19.93	20.07	19.22	0.53	0.58
Stearic acid	C18:0	8.42	8.57	9.44	8.27	7.57	8.65	0.45	0.77
Arachidic acid	C20:0	0.67	0.67	0.51	0.49	0.61	0.54	0.04	0.31
Total SFA		30.69	27.68	31	30.25	29.9	30.01	0.52	0.83
Palmitoleic acid	C16:1	8.61	4.93	7.11	7.92	7.3	7.57	0.73	0.87
Oleic acid	C18:1	23.33	24.33	24.01	22.06	23.69	23.68	0.79	0.91
Eicosenoic acid	C20:1	0.71	0.56	0.63	0.62	0.7	0.62	0.04	0.96
Nervonic acid	C24:1	1.25	1.29	1.3	1.34	1.35	1.31	0.03	0.57
Total MUFA	_	33.9	31.11	33.04	31.94	33.05	33.17	0.81	0.95
Linoleic acid	C18:2 (n-6)	26.97	28.71	28.5	30.19	27.47	28.49	0.53	0.64
Eicosadienoic acid	C20:2 (n-6)	0.67	0.67	0.66	0.68	0.62	0.66	0.03	0.86
DGLA	C20:3 (n-6)	0.59	0.65	0.64	0.7	0.6	0.76	0.04	0.33
Arachidonic acid	C20:4 (n-6)	1.52	1.67	1.62	1.63	1.56	1.68	0.04	0.57
Total Ω-6		29.74	31.7	31.42	31.59	30.25	33.2	0.53	0.56
Alfa-linoleic acid	C18:3 (n-3)	1.53	1.62	1.63	1.62	1.66	1.56	0.05	0.83
Eicosapentanoic acid	C20:5 (n-3)	0.61	0.76	0.57	0.65	0.58	0.74	0.04	0.79
Docosahexaenoic acid	C22:6 (n-3)	1.4	1.42	1.42	1.53	1.44	1.5	0.03	0.38
Total Ω-3	_	3.54	3.8	3.62	3.8	3.68	3.81	0.06	0.45
Total PUF	_	33.28	35.5	35.04	35.39	33.93	36.99	0.54	0.49
MUFA/SFA	_	1.11	1.15	1.06	1.06	1.11	1.1	0.03	0.81
PUFA/SFA	_	1.09	1.31	1.13	1.22	1.14	1.18	0.03	0.95
n-6/n-3	_	8.45	8.37	8.79	8.31	8.3	8.78	0.21	0.97

CON = Basal diet, LE = low energy, LIP = low energy + 0.04% lipase, LYSO = low energy + 0.04% lysolecithin, LIP + LYSO 0.04 = low energy + 0.02% lipase and 0.02% lysolecithin, LIP + LYSO 0.08 = low energy + 0.04% lipase + 0.04% lysolecithin. SEM: Standard error of mean.

## Data Availability

We provided all data in manuscript and haven’t any supplementary data.
